# PSMA-PET/CT in Patients with Recurrent Clear Cell Renal Cell Carcinoma: Histopathological Correlations of Imaging Findings

**DOI:** 10.3390/diagnostics11071142

**Published:** 2021-06-23

**Authors:** Falk Gühne, Philipp Seifert, Bernhard Theis, Matthias Steinert, Martin Freesmeyer, Robert Drescher

**Affiliations:** 1 Clinic of Nuclear Medicine, Jena University Hospital, Am Klinikum 1, 07747 Jena, Germany; falk.guehne@med.uni-jena.de (F.G.); philipp.seifert@med.uni-jena.de (P.S.); robert.drescher@med.uni-jena.de (R.D.); 2 Pathology Division, Institute of Forensic Medicine, Jena University Hospital, Am Klinikum 1, 07747 Jena, Germany; bernhard.theis@med.uni-jena.de; 3 Department of Thoracic Surgery, Leipzig University Hospital, Liebigstraße 18, 04103 Leipzig, Germany; matthias.steinert@medizin.uni-leipzig.de

**Keywords:** PSMA, renal cell carcinoma, PET, PET/CT, histopathology

## Abstract

PET/CT with prostate-specific membrane antigen (PSMA)-targeted tracers has been used in the diagnosis and staging of patients with clear cell renal cell carcinoma (ccRCC). For ccRCC primary tumors, PET parameters were shown to predict histologic grade and features. The aim of this study was to correlate PSMA PET/CT with histopathological findings in patients with metastatic recurrence of ccRCC. Patients with ccRCC who underwent PSMA-targeted PET/CT and subsequent histopathological evaluation of suspicious lesions were included. Specimens underwent immunohistochemical marking. Lesion diameter, volume and tracer uptake were correlated with the extent and intensity of molecular PSMA expression and with clinical findings. Twelve PET-positive lesions of nine patients were evaluated. Eleven ccRCC metastases and one prostate carcinoma were detected histopathologically. Molecular PSMA expression was detected in all lesions, which intensity and distribution did not correlate with PET parameters. PSMA-targeted PET/CT is a feasible tool for the evaluation of patients with ccRCC but cannot reliably predict histologic features of metastases. PSMA may also be expressed in malignant lesions other than ccRCC, leading to incidental detection of these tumors.

## 1. Introduction

Prostate-specific membrane antigen (PSMA) is highly expressed in and widely used for the diagnosis and treatment of prostate cancers [1]. In addition to membranous and cytoplasmic expression in the tumor cells of these neoplasms, it has been shown that PSMA is also expressed in the neovasculature of several other tumor types, being not as specific as its name suggests [2]. Regarding renal cell carcinomas (RCC), studies and clinical experience showed that the imaging accuracy of the established oncologic PET tracer F-18-Fluorodeoxyglucose (FDG) is limited. Since endothelial PSMA expression is seen in the tumor vessels of a majority of clear cell RCC (ccRCC), the idea arose to apply PSMA-targeted tracers in these settings [3]. The first applications of PSMA-targeted PET/CT, using the radioactive tracers Ga-68-PSMA-11 and F-18-DCFPyL, were reported in 2014 and 2015 [4,5]. Due to these promising findings, PSMA-PET/CT is increasingly used for the diagnosis and staging of patients with ccRCC [6,7].

This study was undertaken to evaluate the usefulness of PSMA PET/CT for the detection of metastatic recurrence of ccRCC, and to correlate the results of PET/CT with immunohistochemical findings.

## 2. Materials and Methods

Patients with a history of ccRCC who underwent PSMA-targeted PET/CT in our institution for further evaluation of potential metastases seen on previous imaging (sonography, CT, x-ray), and in which specimens of the suspicious PET/CT lesions were histopathologically assessed, were included in the study.

PET/CT were performed after intravenous injection of Ga-68-PSMA-11, on a Biograph mCT 40 scanner (Siemens Healthineers, Erlangen, Germany). After a low-dose CT for co-registration and attenuation correction with CareDose4D to reduce x-ray exposure (100 kV; slice width 1.2 mm), PET data were acquired from the head to the upper legs. Image reconstruction was performed with HD TrueX software (Siemens Healthineers; parameters: 3 iterations, 24 subsets, no zoom, 5 mm Gaussian filter, matrix 200 × 200). If no recent contrast-enhanced CT was available, a CT of the thorax, abdomen (biphasic) and pelvis was added.

Lesions were considered suspicious for metastasis if they exhibited increased PSMA uptake in comparison to the surrounding tissue and the physiological muscle activity (metabolic criterion), or when a relevant progression in size compared with previous examinations was noted (morphologic criterion). On PET/CT, the maximum and mean standardized uptake values (SUVmax, SUVmean) of suspicious lesions and of the major gluteus muscle were measured. The lesion-to-muscle ratio of SUVmax was calculated. A PSMA expression visual score was used to qualitatively assess tracer uptake in relation to reference tissues: Score 0, below blood pool; 1, equal to or above blood pool and lower than liver; 2, equal to or above liver and lower than parotid gland; 3, equal to or above parotid gland [8]. Maximum lesion diameter and lesion volume were determined on morphologic imaging.

Tissue specimens were obtained by surgery or biopsy and underwent paraffin embedding. In addition to standard histological staining, immunohistochemical PSMA marking with a monoclonal mouse anti-human prostate-specific membrane antigen (clone 3E6; Dako A/S, Glostrup, Denmark) and hematoxylin counterstaining was performed. WHO/ISUP grade was assigned [9]. PSMA expression was evaluated according to the criteria proportion of vessels involved (graded into five categories: 0: none; 1: 1–5%; 2: 6–25%; 3: 26–50%; 4: >50%) and intensity of expression (graded into four categories: 0: none; 1: weak; 2: moderate; 3: strong). Histopathological diagnoses and PET/CT findings were correlated.

### Statistical Analysis

For statistical evaluation, Spearman’s Rho calculations were carried out to evaluate correlations between PET/CT and histopathological findings with the SPSS statistics package (IBM, Armonk, New York, USA; version 24).

## 3. Results

The PET/CT examinations of nine patients (eight men, one woman, age 52–80 years) were included in the evaluation. All patients have undergone primary tumor nephrectomy. Time interval between primary surgery and current PET/CT was 0.4 to 14.2 years (median 4.4 years). PET/CT were performed 74–103 min (median 87 min) after intravenous injection of 221–272 MBq (median 252 MBq) of Ga-68-PSMA-11.

Twelve lesions which were considered suspicious on PET/CT were histopathologically evaluated (Table 1). For a prostate and a bone lesion (lesions 3 and 7) biopsies were performed. All other lesions were surgically resected. In two patients, more than one lesion was evaluated (patient 1: lesions 1–3 and patient 6: lesions 8 and 9). In four out of nine patients, further PSMA-positive lesions suspicious for metastases were identified on PET/CT (osseous, hepatic, lymphonodulary and pulmonary localizations), which were not histopathologically evaluated. Two PET-positive lesions (prostate, pancreatic head; lesions 3 and 11) were not visible on CT alone.

**Figure 1:**
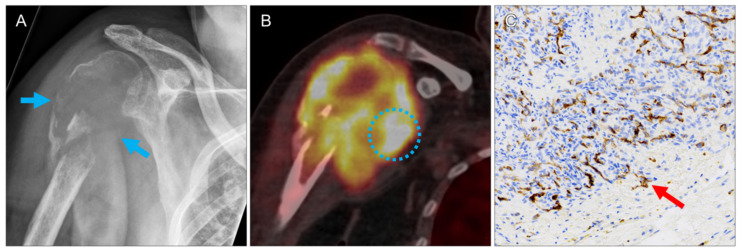
Large osteolytic ccRCC bone metastasis (lesion 8). X-ray shows destruction of the proximal right humerus ((**A**), arrows). PET/CT reveals extensive soft tissue infiltration of the lesion (**B**)), with the highest tracer uptake observed in this study (circle; SUVmax 23.4). Intense PSMA expression on the microvasculature of the mass ((**C**), brown structures, arrow). No intact bone structures were seen in this biopsy specimen.

**Figure 2:**
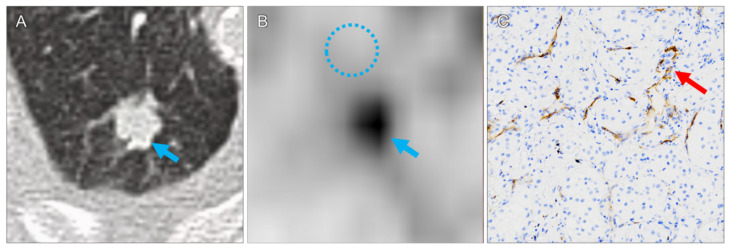
Pulmonary metastasis in the right upper lobe ((**A**), arrow; lesion 6). Due to the low background activity in the lung ((**B**), circle, SUVmax 0.9) the moderate tracer uptake in the tumor is clearly visible ((**B**), arrow, SUVmax 4.2). Moderate microvascular PSMA expression ((**C**), arrow).

**Figure 3:**
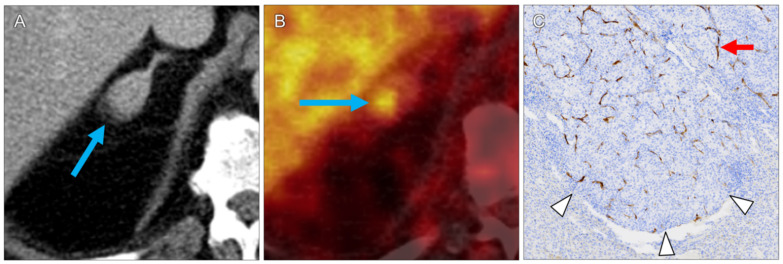
RCC metastasis in the right adrenal gland (lesion 11). Contrast-enhanced CT shows enlargement of the gland ((**A**), arrow). On PET, tracer uptake is highest in its posterior part ((**B**), arrow; SUVmax 7.7). Histology shows invading tumor tissue ((**C**), arrowheads). The brown structures ((**C**), arrow) represent microvascular PSMA expression, which is not seen in the glandular tissue itself.

In all lesions, pathologic correlates to the increased PET tracer uptake were confirmed by histopathology. CcRCC metastases were found in 11 specimens (92%). For these lesions, median diameter and volume were 1.1 cm (range 0.6–8.1 cm) and 0.7 mL (range 0.1–15.2 mL), respectively. Median SUVmax and SUVmean were 3.1 (range 1.2–23.4) and 2.0 (range 0.8–13.7), respectively. The highest SUVmax was measured in a large ccRCC bone metastasis in of the right humeral head (lesion 7, Figure 1). CcRCC metastases in a mediastinal lymph node (lesion 1), in a 1.0 cm upper lobe lung nodule (lesion 6, Figure 2), in an adrenal gland (lesion 10, Figure 3) and in a pancreatic head (lesion 11) showed moderate tracer uptake.

The majority of lung metastases showed low tracer uptake. One nodule had an SUVmax of 4.2 (lesion 6, Figure 2), all other nodules had lower tracer uptake (1.2 to 3.1). Three nodules with an SUVmax below 2.0 (lesions 2, 9 and 12) were considered suspicions not only based on tracer uptake, but also on size progression compared with previous CT examinations.

PSMA visual scores from 0 to 3 occurred. All pulmonary lesions had scores of zero or one; all non-pulmonary lesions had scores of two or higher, including the prostate cancer (lesion 3, score 2).

In five patients the WHO/ISUP grades of the ccRCC metastases were different from the primary tumor (Table 1). Neither microvascular PSMA expression nor intensity of PSMA expression showed a systematic correlation with tumor grade. In all ccRCC metastases, the proportion of PSMA-expressing vessels was above 6%. In two metastases, the proportion was higher than 50%, including the lesion with the highest SUVmax and SUVmean (lesion 7, bone), but also the lesion with SUVmax and SUVmean below 2.0 (lesion 12, lung). Statistical evaluations did not show correlations between PET tracer uptake values and PSMA expression (SUVmax vs. lesion grade: rs(11) = −0.215, *p* = 0.525; SUVmean vs. lesion grade: rs(11) = −0.180, *p* = 0.596; SUVmax vs. proportion of vessels: rs(11) = 0.368, *p* = 0.266; SUVmean vs. proportion of vessels: rs(11) = 0.412, *p* = 0.208; SUVmax vs. intensity: rs(11) = 0.245, *p* = 0.467; SUVmean vs. intensity: rs(11) = 0.288, *p* = 0.391).

**Figure 4:**
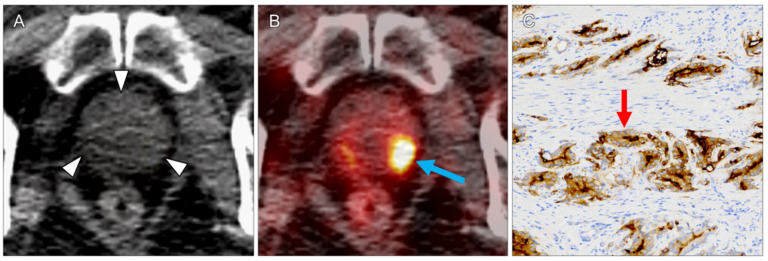
Incidental finding of a prostate carcinoma (lesion 3) in a patient who also had pulmonary and lymph node ccRCC metastases. On non-enhanced CT, the prostate appeared unremarkable ((**A**), arrowheads). PET/CT revealed high focal tracer uptake in the left peripheral zone ((**B**), arrow; SUVmax 9.3). In the biopsy-proven prostate carcinoma, strong PSMA expression on the cytoplasmatic membranes was seen ((**C**), arrow).

One lesion (8%) did not represent a ccRCC metastasis: in the patient who also had a ccRCC lymph node and a lung metastasis (lesions 1 and 2), prostate cancer was detected incidentally (lesion 3, SUVmax 9.3, Figure 4).

## 4. Discussion

During recent years, it has been shown that PET/CT with PSMA-targeted tracers is, in addition to MR and CT imaging, a further imaging option for patients with renal cell carcinoma. The expression of the PSMA ligand in the neovasculature of non-prostatic tumors has been reported as early as 1999 [2]. Regarding renal carcinomas, further studies revealed that it is particularly strong in clear cell RCC: histopathological evaluations of 109 primary tumors showed PSMA expression in 76.2% of clear cell RCC, 31.2% of chromophobe RCC, but none in papillary RCC [3]. PET/CT with PSMA-affine tracers to detect metastatic ccRCC was introduced in 2014 and 2015 [4,5]. In contrast to F-18-flourodesoxyglucose (FDG) PET/CT, which has a limited sensitivity to detect ccRCC metastases, a prospective study of oligometastatic ccRCC patients showed that the PSMA-targeted tracer F-18-DCFPyL PET/CT detected more metastatic lesions than conventional imaging, including CT and MRI [10,11]. However, it was confirmed again that it is not suitable for non-ccRCC, with only 13.7% of those lesions having definitive tracer uptake [12,13].

In the presented study, in all PET-positive lesions PSMA expression was revealed by immunohistopathology. The ccRCC metastases showed variable degrees of microvascular PSMA expression. The highest tracer uptake was detected in a large osteolytic bone metastasis (Figure 1), the second highest in an incidentally detected prostate carcinoma (Figure 4). Differences in the pathological grade between primary ccRCCs and their metastases are a common phenomenon, due to different clones already evident in the primary tumor [14].

For primary ccRCC, a recent study containing 36 patients with preoperative PET/CT and histopathological correlation reported that the PET/CT parameter SUVmax in the tumor can discriminate WHO/ISUP grade (3/4 from 1/2) and adverse pathology (tumor necrosis or sarcomatoid/rhabdoid features) with high sensitivity and specificity [15]. For the ccRCC metastases in our cohort, besides the bone metastasis, all SUVmax values were below 10, whereas in the mentioned publication regarding primary tumors, much higher SUVmax cutoff values of 16.4 and 18.5 were reported for differentiation of WHOS/ISUP grade and adverse pathology, respectively.

We could not find a correlation between PET/CT parameters (SUVmax, SUVmean, lesion-muscle-ratio) and histopathological findings (extent and intensity of molecular PSMA expression). PET/CT examinations in general have technical limitations regarding the uptake quantification of small pulmonary nodules, which is impaired further by the respiratory motions of the lung bases. In small nodules, because of the limited physical resolution of PET, uptake values may be artificially low due to partial volume effects (SUVmax is the uptake of the hottest voxel within a defined volume of interest, but two voxels may already be larger than the nodule) [16]. In a study evaluating PSMA PET/CT for whole-body staging of renal cell carcinoma, all metastases considered PET-negative were subcentimeter lung nodules (mean diameter 0.7 cm, range 0.5–0.9 cm) [17]. Most small pulmonary nodules in our study had low SUV values but were still visible on PET against the low background activity in the lungs (Figure 2). The three cases in which the recommendation for surgery was based not on tracer uptake alone, but also on nodule progression on CT, reaffirms the advantages of hybrid imaging, and that all available images should be taken into account. In this setting, even extremely small lung nodules with low PSMA uptake should be considered suspicious. A further reason may be histopathological sampling errors. Not the whole tissue specimen can be histopathologically evaluated, and even in a small metastasis PSMA expression and intensity is inhomogeneous. The largest lesion with the highest uptake (lesion 7) had the highest proportion of vessels expressing PSMA, and also a high PSMA expression intensity, suggesting that no significant correlations between PET/CT parameters and histopathology findings were found due to a combination of two factors: PSMA expression in a lesion is inhomogeneous on a millimeter-to-submillimeter level, and PET resolution is limited to several millimeters, which leads to averaging effects in a voxel and the resulting SUV_max_ and SUV_mean_ measurements.

In order to standardize the interpretation of PSMA-PET/CT examinations and to increase comparability and reproducibility in clinical trial, consensus interpretation guidelines were developed by an international panel [8]. The mediastinal blood pool activity as well as parotideal and liver tracer uptakes have been proposed as references, leading to a qualitative score to describe the degree of PSMA avidity of a lesion. In our cohort, the only lesion with a visually higher uptake than the parotid gland was the large metastasis in the humerus (lesion 3). Most lesions were classified as PSMA uptake scores 1–2, whereas the three smallest pulmonary lesions had a score of 0 (below blood pool). The visual, qualitative score reflected well the quantitative SUV measurement results, but a score of 0 did not rule out metastases.

A general clinical relevance of PSMA-PET/CT can be assumed, since a retrospective evaluation of Ga-68-PSMA-PET/CT examinations in RCC patients showed that PET/CT directly changed management in 42% of cases, 87.5% of PET-positive patients had ccRCC and one of eight ccRCC was PET-negative [18]. PET/CT with PSMA-targeting radiopharmaceuticals has the potential to assess the treatment response in ccRCC patients receiving tyrosine–kinase inhibitor or anti-angiogenic treatments [19,20]. It may even open a way to PSMA-based radioligand therapies (RLT) in ccRCC patients with high uptake tumors and metastases [21].

As with other tumors, dedifferentiation of ccRCC leads to a loss of specific characteristics: highly differentiated neuroendocrine tumors show strong somatostatin receptor (SSR) expression, visualized on DOTATOC-PET/CT, whereas non-differentiated neuroendocrine tumors (NET) have a low DOTATOC and a high glucose (FDG) uptake on PET/CT. In ccRCC, lesions with sarcomatoid differentiation, which is an adverse prognostic factor and occurs in 5% of cases, show high glucose and low PSMA uptake, whereas non-sarcomatoid lesions show high PSMA and low glucose uptake [22].

In this study, no lesions with non-specific tracer uptake were seen, but non-tumoral PSMA uptake on PET/CT has been described [23]. Detailed correlation with morphologic imaging and clinical symptoms remains a necessity.

Limitations of the study are the low number of lesions which could be histopathologically evaluated, particularly in non-pulmonary localizations. We did not have the possibility to compare findings of metastases with those of primary tumors, because no patient underwent PSMA-PET/CT before nephrectomy. The existence of PSMA-negative ccRCC metastases cannot be ruled out.

In conclusion, it was confirmed that PSMA-targeted PET/CT is a valuable and reliable tool for the evaluation of patients with suspected ccRCC metastases. It must be kept in mind that PET/CT may also be positive in malignant lesions other than ccRCC and prostate cancer, leading to incidental detection of these tumors. This may decrease the specificity of the examination for a particular entity, but facilitates early diagnostic and treatment in some cases.

## Figures and Tables

**Table 1:** Characteristics of histopathologically evaluated lesions.

Lesion		PET/CT Findings						Histopathology Findings			PSMA Expression	
No.	Localization	Lesion Diameter (cm)	Lesion Volume (mL)	SUV Max	SUV Mean	Lesion- Muscle- Ratio	PSMA Visual Score	Diagnosis	Primary Tumor Grade ^1^	Lesion Grade ^1^	Proportion of Vessels ^2^	Intensity ^3^
1	mediastinal lymph node	1.8	1.2	5.9	4.4	7.4	2	ccRCC	2	2	3	2
2	lung, right upper lobe	0.7	0.2	1.2	0.8	1.5	0	ccRCC	2	2	2	2
3	prostate	1.2	0.9	9.3	6.3	11.6	2	PC	-	-	-	3
4	lung, right lower lobe	1.5	1.8	2.8	1.8	2.5	1	ccRCC	3	4	2	2
5	lung, left lower lobe	1.1	0.7	1.7	1.3	2.8	1	ccRCC	2	3	2	2
6	lung, right upper lobe	1.0	0.5	4.2	2.9	5.3	1	ccRCC	4	2	3	2
7	right humerus	8.1	15.2	23.4	13.7	26.0	3	ccRCC	3	2	4	3
8	lung, right lower lobe	1.1	0.7	3.1	2.0	3.9	1	ccRCC	2	2	3	2
9	lung, right lower lobe	0.9	0.4	1.6	1.1	2.0	0	ccRCC	2	2	3	2
10	right adrenal gland	1.2	0.9	7.7	4.9	7.7	2	ccRCC	3	3	3	3
11	pancreatic head	1.3	0.9	6.1	4.3	6.8	2	ccRCC	1	1	2	1
12	lung, left upper lobe	0.6	0.1	1.9	1.6	3.2	0	ccRCC	2	3	4	3

Abbreviations: ccRCC—clear cell renal cell carcinoma, PC—prostate carcinoma. ^1^ WHO/ISUP grade; ^2^ categories: 0, none; 1: 1–5%; 2: 6–25%; 3: 26–50%; 4: >50%; ^3^ categories: 0, none; 1: weak; 2: moderate; 3: strong.
